# Chromatography-Free Purification Strategies for Large Biological Macromolecular Complexes Involving Fractionated PEG Precipitation and Density Gradients

**DOI:** 10.3390/life11121289

**Published:** 2021-11-24

**Authors:** Fabian Henneberg, Ashwin Chari

**Affiliations:** 1 Department of Structural Dynamics, Max Planck Institute for Biophysical Chemistry, Am Fassberg 11, D-37077 Göttingen, Germany; fabian.henneberg@mpibpc.mpg.de; 2 Research Group for Structural Biochemistry and Mechanisms, Max Planck Institute for Biophysical Chemistry, Am Fassberg 11, D-37077 Göttingen, Germany

**Keywords:** protein purification, PEG precipitation, protein precipitation, chromatography-free protein purification, sucrose density gradient centrifugation, 20S proteasome, 26S proteasome, fatty acid synthase, immunoproteasome, buffer optimization, monolithic columns, macromolecular complexes

## Abstract

A complex interplay between several biological macromolecules maintains cellular homeostasis. Generally, the demanding chemical reactions which sustain life are not performed by individual macromolecules, but rather by several proteins that together form a macromolecular complex. Understanding the functional interactions amongst subunits of these macromolecular machines is fundamental to elucidate mechanisms by which they maintain homeostasis. As the faithful function of macromolecular complexes is essential for cell survival, their mis-function leads to the development of human diseases. Furthermore, detailed mechanistic interrogation of the function of macromolecular machines can be exploited to develop and optimize biotechnological processes. The purification of intact macromolecular complexes is an essential prerequisite for this; however, chromatographic purification schemes can induce the dissociation of subunits or the disintegration of the whole complex. Here, we discuss the development and application of chromatography-free purification strategies based on fractionated PEG precipitation and orthogonal density gradient centrifugation that overcomes existing limitations of established chromatographic purification protocols. The presented case studies illustrate the capabilities of these procedures for the purification of macromolecular complexes.

## 1. Introduction

Cells and organisms constantly perform a multitude of tasks for their survival. These include the conversion of the many forms of physical and chemical energy in their surroundings to chemical molecules to support growth. Spent, expended, and erroneous molecules are constantly monitored and expelled from the cell/organism. In this manner, a homeostatic balance is upheld, which sustains life [1]. The key molecules enabling such chemical transformations are nucleic acids (RNA and DNA which are linear chains of nucleotides), proteins (linear chains of amino acids), carbohydrates (structural functions in cell walls and energy storage), and lipids (constituents of membranes, membrane tethering-, and signaling molecules) [2]. While all biological molecules consist of these relatively simple building blocks, they can exhibit an almost endless diversity in the substrates they work on, the chemical reactions they enable, catalyze, and conduct. This functional diversity contrasts with the common building blocks of biological molecules and therefore an additional component, in addition to composition, has to account for their functional abilities. Consequently, basic research efforts in the life sciences aim at investigating the mechanisms, by which cells uphold homeostasis. In addition, as cellular homeostasis depends on a finely balanced accurate functioning and cooperation of biological molecules, their mis-function is a central cause for human disease. Thus, detailed investigations into the mechanisms of biological molecule function are essential for the study of disease etiology [3].

A major step forward in this quest was the sequencing of entire genomes of a variety of model organisms [4]. The pinnacle of genome sequencing efforts certainly was the achievement of sequencing the whole human genome, which was a milestone for molecular biology [5,6]. Since then, the majority of all protein-coding genes are known and can be described in the context of their coding sequences. Deciphering genomes, in particular the human genome, has also provided the basis for addressing the next challenges in the life sciences: firstly, how is the information encoded in the genetic blueprint expressed and translated to generate complex living organisms? Secondly, how do these myriads of molecules interact and communicate with each other in biomolecular interaction networks to perform the complex tasks essential to cellular homeostasis? 

To tackle the first challenge, namely the deciphering of gene expression profiles and patterns, great strides and technical advances have been made in the field of transcriptomics [7]. RNA sequencing was propelled by next-generation sequencing techniques, allowing to monitor the continuously changing cellular transcriptome at a certain time point [8]. These studies have led to the realization that multiple layers of complexity are introduced post-transcriptionally to the DNA-encoded genetic blueprint. These include alternative splicing events [9], editing of bases in messenger RNA [10], and regulatory incidents in the translation of mRNA to protein [11]. While many questions have arisen and additional complexity layers have been uncovered as a consequence of these studies, these have laid the foundation to tackle the questions how biomolecular interaction networks are wired and how they mediate cellular homeostasis. For this purpose, genome-wide studies have been performed, where all open reading frames of a given organism are tagged by a short sequence motive that can be used in pulldown experiments. In particular, the tandem affinity purification (TAP)-tagging strategy, originally pioneered for the yeast *S. cerevisiae* but later adapted to mammalian cell lines, has been instrumental in these endeavors [12,13]. As the TAP-tagged proteins are purified in a native state from endogenous pools, and the TAP purification technique makes use of mild elution conditions, it allows for the co-purification of proteins interacting with the TAP-tagged bait [14]. Bait and co-purified proteins can be identified by SDS-PAGE, in-gel proteolysis, and subsequent tandem mass spectrometry (MS/MS) [15]. Here the readily available databases of whole genomes and proteomes are yet again enormously helpful. Proteolytic fragments of proteins can be blasted against these databases allowing for their identification with high accuracy [16].

Gavin and co-workers employed systematic whole genome TAP-tagging, purifications, and MS/ MS in the baker’s yeast *S. cerevisiae* to systematically identify biological interaction networks within this organism [17]. The outcome was the realization that most proteins do not fulfill their designated tasks in isolation, but are rather incorporated into macromolecular complexes of 12 components on average. It is these higher-order assemblies that maintain cellular homeostasis, and strikingly, most of the observed complexes either were unknown or contained at least one or many new components. The biological interaction networks could be assigned to nine different functional categories. In addition, several proteins with unknown functions could be annotated, and some proteins with known functions could be linked to additional functional contexts. This study, along with numerous genome-wide systematic yeast two-hybrid studies, tagged purifications, and tandem MS identifications have massively increased our understanding of the functional interactome within cells and organisms [12,17,18]. These insights not only are important for basic research to provide insight into the basis of life, but also are of paramount interest therapeutically as many of these macromolecular complexes are potential high-value drug targets for various diseases. Additionally, cellular components have evolved to perform all chemical reactions within aqueous environments, a balanced temperature profile, with high efficiency and specificity. In the search for sustainability, these processes are sought to be mirrored by chemical industry, which often uses harsh conditions and chemicals to perform the same reactions.

## 2. Pitfalls and Difficulties in the Investigation of Macromolecular Complex Function

The aforementioned genomic and proteomic studies have firmly highlighted the importance of large assemblies in maintaining cellular homeostasis. The high number of newly discovered macromolecular complexes and the detection of additional interacting proteins for known complexes has stressed that our insights into the biomolecular interactome remain sparse. This most likely has a root in technical limitations in the methods used to purify biological macromolecules in general, and macromolecular complexes in particular. We will outline some of these pitfalls and difficulties in the following. A first step towards understanding the function of macromolecular complexes is to unequivocally establish their composition. However, precisely this seemingly simple task is complicated by the fact that the composition of the biological interactome critically depends on the employed purification conditions [19]. On the one hand, the response to cellular growth conditions and extracellular cues strongly, functionally influences the composition of macromolecular complexes [17,20]. On the other hand, using different extraction conditions for cells expressing the same tagged protein can strongly impact the composition of purified macromolecular complexes [21]. In addition, it is common knowledge that buffer conditions employed to purify complexes have an impact on their integrity [22]. Aside from the conditions utilized for cell growth, the extraction of biological molecules, and the employed purification conditions, the precise position of the affinity tag may have an impact on the co-purified constituents. This can arise from affinity tags influencing the interaction amongst neighboring subunits in macromolecular complexes preventing the formation of the native protein complex [23]. It is also possible that the interaction of tag and the cognate affinity chromatographic support sterically dissociates the complex, especially when the interaction between tag and support is stronger than that of the subunits amongst each other. Moreover, transiently interacting proteins are difficult to capture as they are present only for a limited period and their interaction is labile compared to the tag-chromatographic support association [17].

**Figure 1:**
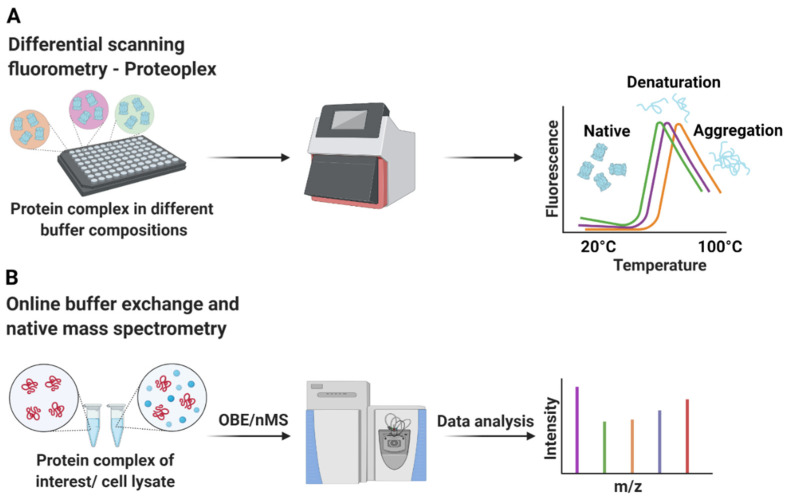
Buffer conditions indicated by different colors for protein complexes can be optimized by differential scanning fluorometry (DSF) (**A**) and online buffer exchange (OBE) in combination with native mass spectrometry (nMS) (**B**). The melting temperatures and unfolding behavior of protein complexes determined in thermal shift assays are employed to quantify their stability in various buffer formulations. Online buffer exchanges provide a platform to efficiently screen various buffer conditions. The structural integrity of the protein complexes is then analyzed by native mass spectrometry (nMS).

To assess their function, an in-depth functional characterization of these biological interaction networks in a purified state is indispensable. The same is true when developing pharmaceuticals tailored to counteract, or modulate the function of macromolecular complexes. Often, it is imperative to experimentally determine their 3D architecture to great detail and at high-resolution, to fully understand the function and address mechanistic questions. Favorably, in this regard, the recent years have seen a substantial advance in the methodology to determine 3D structures of macromolecular complexes of ever-increasing size and complexity [24,25,26,27,28,29]. Both approaches depend on biological assemblies purified to homogeneity, in an intact manner, of high quality and activity. Considering the strong dependence of biological macromolecule preparations on buffer composition, it is not surprising that great attention is paid to the careful optimization of buffer formulations. While this traditionally is a tedious task that is strictly trial-and-error, and cumbersome, recent technical developments have sped up the processes and increased the throughput (Figure 1).

To assess the structural integrity of macromolecular complexes, several methods are employed. The stability of macromolecular complexes can be interrogated by employing thermal shift assays in a purified state. These techniques provide a useful platform to optimize buffers conditions to increase the thermodynamical stability of the macromolecular complexes, in a high throughput screening approach (Figure 1A) [30,31,32] (Stark et al., 2016, WO2013034160). Recently, to complement thermal shift approaches, online buffer exchange (OBE) coupled with native mass spectrometry (nMS) has been implemented (Figure 1B) [33]. This method is geared to the analysis of tertiary and quaternary structure to obtain information about macromolecular complex integrity, and to monitor stabilizing buffer conditions. All mentioned methods can be used to increase the stability of macromolecular complexes by adjusting buffer conditions. However, if the integrity of these assemblies has been compromised in the course of purification, there is usually no other outcome than going back to the source material, re-applying the optimized buffer conditions, and re-purification. OBE-nMS might be beneficial over thermal shift assays in this regard as this method can be applied directly to cell lysates containing the sample-of-interest to provide insight into the native protein complex composition in the cell [33]. Irrespective of the method used for assessment, the general outcome of stability optimization approaches is that most, if not all macromolecular complexes have a very narrow window of stability in terms of the chemical nature of buffer, ionic strength, additives, and pH.

## 3. Pitfalls of Chromatographic Purification Strategies for Large Macromolecular Complexes

**Figure 2:**
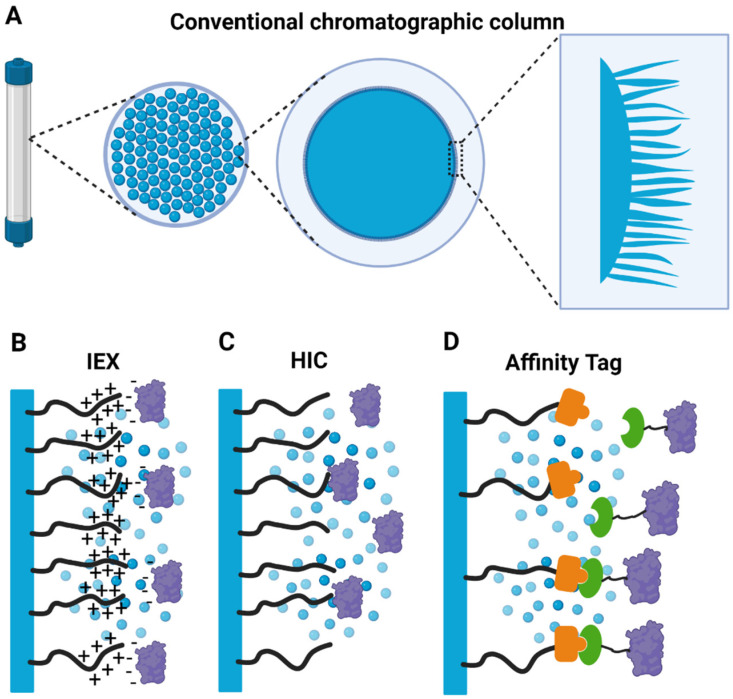
Conventional chromatographic media usually consists of porous particles like dextran and agarose beads as stationary phases (**A**). The beads are modified with a coating layer to provide functionality for ion exchange chromatography (**B**), hydrophobic interaction chromatography (**C**), or affinity tag purification (**D**).

The absolute requirement of both structural and functional characterization of macromolecular complexes dictates that they are purified in high-quality and homogenous. Based on the successful application to both chemical molecules and single biological macromolecules, chromatographic strategies are commonly used to purify macromolecular complexes, [34,35,36,37]. As discussed above, once biological assemblies are subject to either partial-, or complete denaturation, and subunits dissociate, no recovery is possible from the denatured or dissociated state. As biological macromolecules are stable in aqueous solutions, chromatographic procedures of macromolecular complexes are mostly normal phase operations. Traditionally, techniques such as ion-exchange, hydrophobic interaction, chromato-focusing, affinity, and size-exclusion chromatography have been used [35,38,39,40] (Figure 2B,C). More recently, multimodal chromatographic procedures have been incorporated into the macromolecule- and biological assembly purification toolbox. 

However, with the advent of widespread genomic and proteomic information, along with extensive development of molecular biology techniques [41], most purification schemes rely on (tagged-) affinity chromatography and subsequent size-exclusion chromatography (Figure 2D) [42]. While this has the advantage that purification methods have been simplified substantially and less experimental experience is required, some caveats have to be mentioned.

As described in the previous paragraph, the narrow stability window of buffer formulation in which macromolecular complexes are stable has a substantial impact on the usage of chromatographic methods for protein purification of large biological assemblies. The optimization of buffer formulations in chromatography firstly aims for efficient interaction between the protein and the stationary phase, and secondly for the selectivity of the applied purification chromatographic technique. For instance, ion-exchange chromatography relies on pH that increases the net charge of the protein, and low ionic strength for an efficient binding of the sample to the ion-exchange media (Figure 2B). Conversely, for elution, buffers with high salt concentrations resulting in high ionic strength, or large pH changes inducing an altered charge distribution on the protein surface, are used [38]. Similar, but distinct frameworks exist for all other chromatographic methods including hydrophobic interaction-, chromatofocusing-, multimodal-, and affinity chromatography [43]. These could coincide with the narrow window of buffer formulation required to maintain the stability of macromolecular complexes, on the other hand, they could also lead to the loss of loosely bound subunits, and/or dissociation of the whole macromolecular complex.

**Figure 3:**
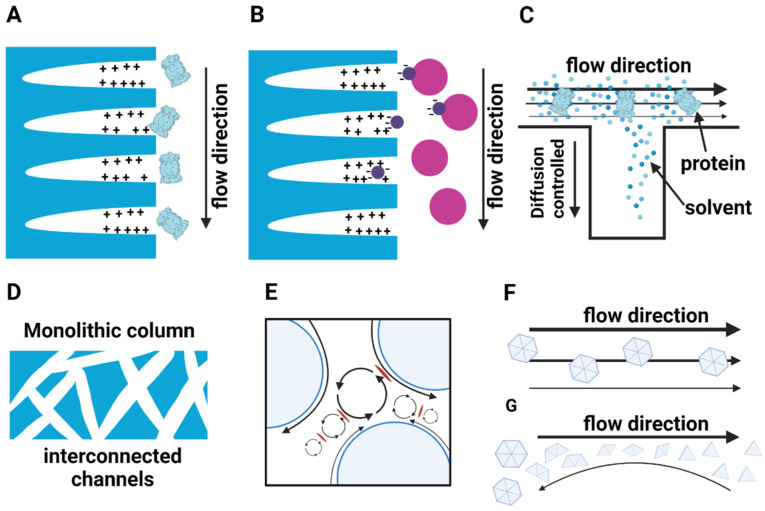
Different pore sizes exist which determine the accessibility of macromolecules to the functional groups of the chromatographpic support. If the macromolecular complex size exceeds the pore size, the macromolecular complexes cannot reach the functional groups (**A**). Low-affinity subunits of macromolecular complexes which are attracted to the functional groups of the stationary phase dissociate and can cause the macromolecular complex to disintegrate (**B**). The mass transport into the pores is determined by diffusion which highly depends on the flow rate and causes concentration gradients within the pore (**C**). The monolithic stationary phase is highly interconnected by large pores resulting in a convective mass transport avoiding concentration gradients (**D**). Two main different shear stresses exist in chromatography. Anti-parallel forces resulting from opposing flows cause turbulent shear (**G**) and laminar shear forces are the result of different flow velocities inside a pore (**F**). In the void volume between the porous particles composing the chromatographic matrix, turbulent shear forces caused by eddy vortices occur causing the macromolecular complexes to disintegrate (**E**).

An additional aspect to keep in mind with regards to chromatographic purification of large macromolecular complexes is that, traditionally, chromatographic media consist of crosslinked agarose-, or Sephadex^®^ beads (Figure 2A) [43]. These supports are then chemically modified on hydroxyl groups with functional groups, conferring them with the property which is to be exploited in the respective chromatographic technique. The extent of crosslinking defines pore sizes which range from 10 to 125 nm and the chemically introduced functional groups of chromatographic media typically reside within these pores (Figure 3A,B) [43]. 

Convective flow dominates mass transport in the void volume between the porous particles. The mass transport inside the porous particles, which determines the access to the sorbent matrix, is additionally controlled by diffusion, due to the small diameter of the pores (Figure 3C) [44]. Transmission electron microscopy data analysis of CM Sepharose revealed a low connectivity of pore paths. Moreover, long pores and narrow pore sizes hinder an effective transport of molecules [45]. This has a profound impact on the purification of large biological molecules and macromolecular complexes in particular. The diffusion-controlled transfer into and out of the pores of conventional chromatographic supports results in macromolecular complexes with their large molecular weights (especially when in excess of 500 kDa) generally purified at lower concentrations, compared to their small protein counterparts [44]. Another inevitable consequence of chromatographic purification of large macromolecular complexes being diffusion-controlled is that the speed of each purification step is also considerably slower in comparison to smaller molecules [46]. Especially in early purification stages, this exposes samples to proteases or nucleases for prolonged times, which is counteractive to sample homogeneity and integrity. The low sample concentrations during chromatography also might result in concentrations which lie below a certain stability threshold. Especially transiently bound, peripheral and regulatory subunits exhibit low affinities to the respective cognate complexes. Should prolonged stages exist where the concentrations are low, falling below the dissociation constant of the interaction of these subunits with the given macromolecular complex, these are bound to stochastically dissociate from the macromolecular complex [43,47]. Furthermore, the pores provide large surfaces for non-specific interactions with the stationary phase which are enhanced by the low concentrations of the macromolecular complex in the course of purification and the residence time inside the pore. These non-specific interactions may be more avid than the weak forces by which peripheral and transient subunits interact with the biological assembly. The bead structure of chromatographic material additionally creates eddy vortices in the void compartment [48] (Figure 3E). Anti-parallel forces arising from opposing flows generated by eddy vortices give rise to high shear forces on proteins eventually causing their dissociation (Figure 3G) [49,50].

The recent years have seen technological developments to counteract the drawbacks of conventional chromatographic media [51,52]. These include monolithic columns which are formed by a single block defining the dimensions of the columns and can consist of a variety of support materials [53]. These monoliths avoid the detrimental properties of porous media as they rely on interconnected convective channels. As a consequence, these columns can be operated with high flow rates, while simultaneously providing superior binding capacities for the purification of large biomolecular structures [54,55,56,57]. The resulting concentrations of large macromolecular complexes in the course of purification can thus remain high avoiding the pitfalls arising from low sample concentrations. The occurring laminar shear forces are also less harmful to macromolecular complexes compared to the turbulent shear forces experienced in porous particle chromatography (Figure 3F). The monolithic columns provide a significant step forward to preserve the structural integrity of macromolecular complexes. Another notably favorable development is the widespread application of magnetic beads [58]. As suggested by the name, these chromatographic supports have a dense paramagnetic core that can be exploited in purification and recovery. As all functional groups are on the surface of the beads and no pores exist, most of the adverse effects associated with these properties of conventional chromatographic media can be entirely avoided [58]. In addition, the surfaces can be efficiently passivated to minimize adverse surface binding effects. Despite these promising new developments in chromatographic separations to name a few, some unfavorable aspects remain and are bound to persist: the absolute necessity to interact with solid supports, however small, and the application of buffer formulations which are primarily geared towards promoting binding to the solid support and elution therefrom. 

## 4. Development of Chromatography-Free Purification Procedures

While chromatographic purification schemes undoubtedly have their benefits in protein purification, for the reasons brought forward above, schemes which entirely alleviate the use of solid supports might be beneficial. Thus, particularly for large macromolecular complexes, purification schemes should ideally exclusively occur without immobilization, should employ buffer formulations that are within the empirically determined stability window for the biological assembly of interest, and maintain high concentrations throughout the entire purification procedure. In the last decade, we have spent substantial efforts to develop generally applicable purification procedures based on these principles to purify large macromolecular complexes (especially such in excess of 500 kDa in size). Considering these pre-requisites, we surmised that simple, selective, and fractionated precipitation techniques should be able to meet these requirements. Owing to the hydrodynamic radius and size of large biological assemblies, we reasoned that size separation on density gradients should be an ideal orthogonal purification method. The question then arises which precipitation agent and procedure to use? Salting-out procedures did not appear lucrative as these would raise the ionic strength for the biological assemblies and induce conditions, which might lie outside of their respective window of stability [30]. In contrast, fractionated precipitation using the non-ionic polymer polyethylene glycol (PEG) appeared promising and suited the pre-requisites raised above.

It should also be noted that, formally speaking, PEG can also be utilized as a partitioning agent for the purification of protein samples Albertsson was the first person to apply PEG to selectively partition proteins in a PEG-salt or PEG-dextran suspensions. He could adjust the partition coefficient of proteins between the two phases by varying the salt composition [59]. Based on his findings the field of aqueous two-phase systems (ATPS) was founded. ATPS provide a platform to gently purify proteins in a batch like manner. Nowadays, a large diversity of ATPS are available which are optimized for manifold applications, and have already been exhaustively reviewed [60,61] and in references therein. The detailed discussion of ATPS would exceed the scope of this review and therefore will not be further discussed here. However, pertaining to the discussion in this review, it is worth mentioning that some proteins could not be retained in one of the two phases of ATPS, since they tend to precipitate already at low PEG concentrations needed for phase separation. This drawback of ATPS has already been reported very early on in the literature [62].

The phenomenon of protein precipitation by polymers was then intensively investigated by Polson [63]. Compared to other precipitation techniques with ethanol and polymers like dextran, PVE, PVP, and NPE, PEG does not denature proteins at ambient temperatures [64,65], and therefore was deemed to be the most suitable polymer to precipitate proteins [63]. The molecular basis for PEG precipitation is explained with two different theories—the theory of excluded volume and the attractive depletion theory. Recent in silico simulations of PEG precipitation use the attractive depletion theory as a framework for their models [66]. The mechanism of protein precipitation, in a sense, resembles principles exploited in hydrophobic interaction chromatography (HIC) [67]. Water molecules located close to hydrophobic areas of the protein are well ordered. In the course of protein precipitation, the protein structure slightly changes and hydrophobic areas of protein can interact with each other. This leads to a migration of well-ordered, localized water molecules to the disordered bulk solvent. The gain of entropy makes precipitation thermodynamically favorable.

**Figure 4:**
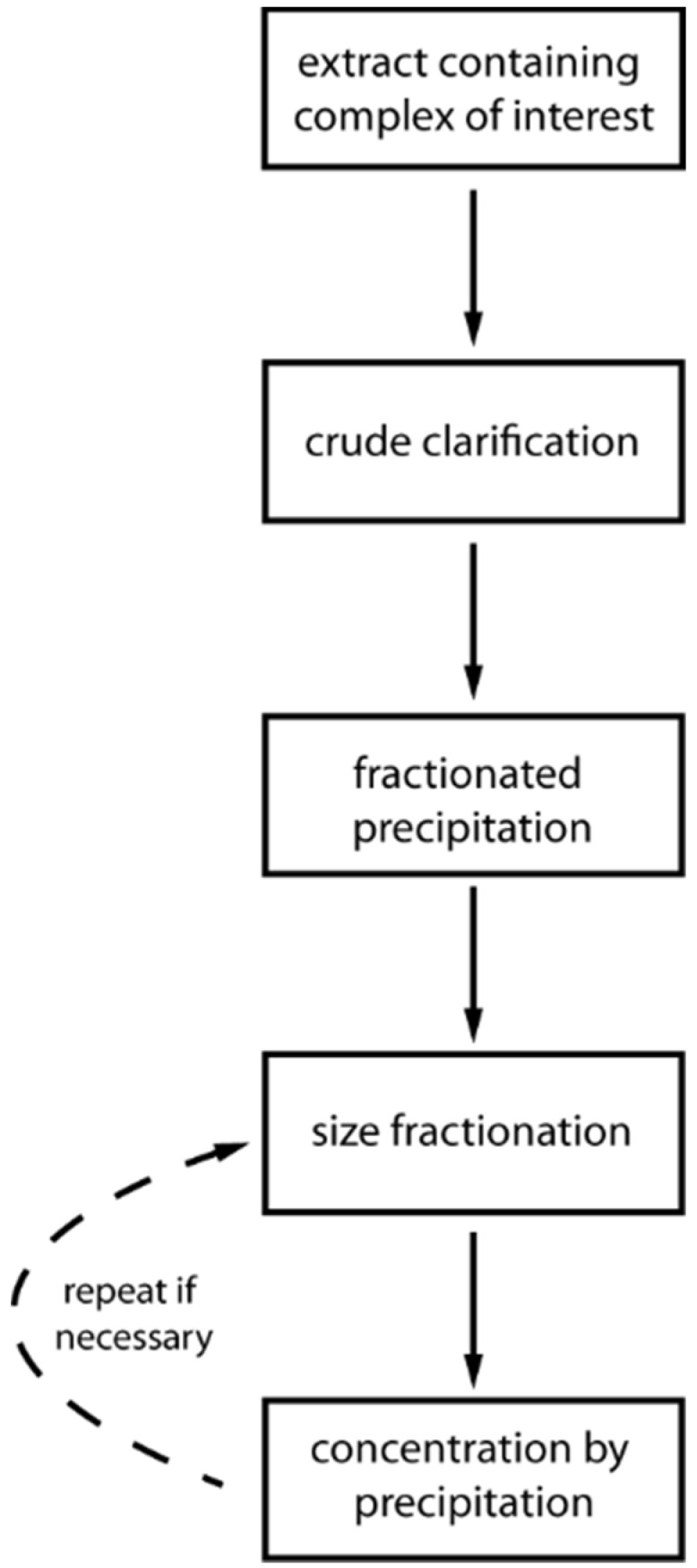
Generalized flowchart of the purification protocol based on the combination of PEG precipitation and sucrose density gradient centrifugation.

Atha and Ingham analyzed the precipitation properties of several different proteins [64]. According to their evaluation, the solubilities of proteins decrease exponentially with increasing concentrations of PEG. The buffer composition of the PEG solution used for precipitation can be adjusted, with the ionic strength and pH altering the PEG concentration needed for protein precipitation. The only distinct incompatibility of buffering species or salts lies in the immiscibility of phosphate and sulfate salts above a certain concentration with PEG solutions, which then form two aqueous solvent phases [68]. As discussed above, this immiscibility forms the basis for ATPS applications. PEGs exist in different molecular weights: higher molecular weight PEGs (>1000 Da) precipitate proteins at a lower concentration than low molecular weight PEGs (<1000 Da). Co-solutes can influence the protein precipitation behavior by PEG substantially [69]. The PEG concentration needed for precipitation of a given protein or macromolecular complex from complex mixtures like cell lysates is difficult to predict and has to be determined empirically. The combination of PEG precipitation with sucrose density centrifugation, as mentioned earlier, is a powerful method to purify large macromolecular complexes orthogonal to PEG precipitation. Sucrose density centrifugation offers the possibility to separate precipitated proteins according to their density and radius of gyration because protein precipitation is not always selective for one protein. Macromolecular complexes are enriched in a fraction of the gradient where the centrifugal force acting on the macromolecular complexes equals the experienced friction in the gradient. Therefore, the dilution of a sample during sucrose density centrifugation is smaller compared to chromatographic purification. Additionally, in contrast to liquid chromatography, the biological assemblies remain in a high-density kosmotropic osmolyte environment resembling their intracellular environment during centrifugation. A flowchart of a typical, generic procedure utilizing the combination of PEG precipitation and density gradient centrifugation is depicted in Figure 4, which has been patented (Chari et al., 2021, WO2017211775).

In the following, we will present some case studies where the generic procedure outlined in Figure 4 is adapted to some macromolecular complexes of different sources and sizes. The benefits compared to conventional chromatographic purification will be mentioned when appropriate.

### 4.1. Case Study 1: Human Constitutive 20S Proteasome

The capabilities of PEG precipitation in combination with sucrose density centrifugation for successful and mild protein purification were systematically evaluated for the proteasome. The proteasome is a macromolecular complex indispensable to the preservation of cellular homeostasis, as it has a key role in protein degradation. Approved anti-cancer therapies making use of the proteasome as a drug target validate the need for detailed structural analysis of this complex. Depending on the function, different proteasome complexes exist: the 20S proteasome, also known as the core particle (CP), is composed of 28 subunits, has a size of 700 kDa, and provides the proteolytic activity for all proteasome holoenzymes. For high throughput screening campaigns addressing the specific inhibition of the 20S proteasome proteolytic activity with novel inhibitors, the macromolecular complex must be purified reproducibly in a high sample quality to guarantee reliable crystallization, and to obtain meaningful kinetic data. Furthermore, high amounts of protein are necessary for an efficient screening process setting the boundary conditions to be fulfilled by any new purification protocol. Firstly, 20S proteasomes, owing to their complexity and multi-step assembly pathway [70,71], need to be purified from human sources, as viable protocols for recombinant expression did not exist until very recently [72]. Secondly, no affinity tag should be used which might affect the structural integrity and/or activity of the 20S proteasome complex. Thirdly, the purification needs to be quick to reduce the abundance of heterogeneity resulting from prolonged persistence in cell lysates. Fourthly, the yield of purified protein needs to be high enough to allow for the realization of high throughput crystallization campaigns. 

**Figure 5:**
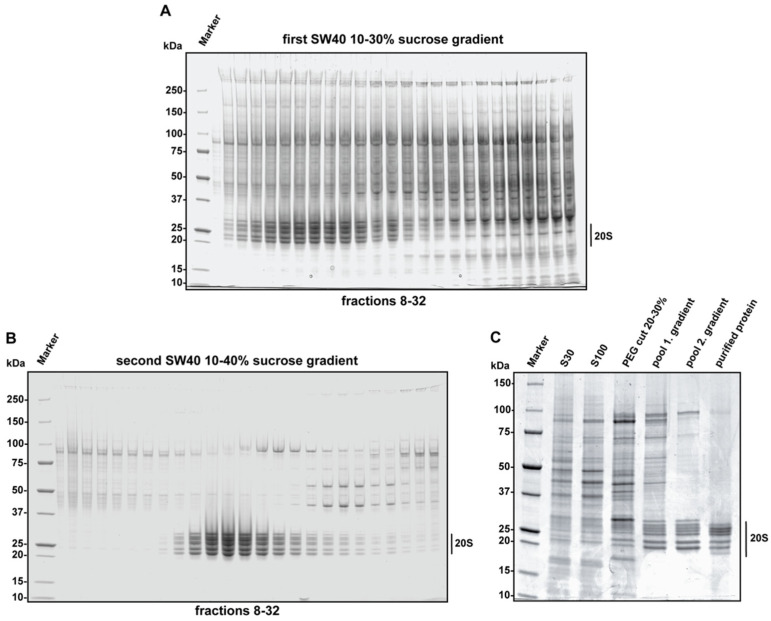
The optimal PEG400 concentrations for human 20S proteasome precipitation are tested empirically. The PEG cut 20–30% (*v/v*) contains the majority of human 20S proteasome and was separated on a sucrose gradient (**A**). 20S proteasome containing fractions were precipitated and loaded on a second sucrose density gradient for further purification (**B**). All steps of the purification procedure are visualized on an SDS-gel (**C**).

The initial steps of protein purification do not differ from well-established purification protocols. Cells are lysed with an appropriate method and the osmolyte concentration is increased to resemble the cytoplasmic environment. Additionally, pH and salts are adjusted according to results obtained from Proteoplex measurements ensuring the optimal stability of the 20S proteasomes [30]. The crude extract is then cleared by centrifugation to remove cell debris. An impurity commonly observed in the course of purification is ribosomes. Therefore, a centrifugation step at 100,000× *g* was introduced to remove the majority of ribosomes before fractionated PEG precipitation. The PEG, dissolved in the stabilization buffer, is slowly added to the clarified extract at room temperature and mixed for 30 min. The precipitate is then collected by centrifugation, the retrieved pellet resuspended in a suitable buffer, and the supernatant is subjected to further precipitation using a higher PEG concentration. The optimal PEG concentration range and molecular weight for fractionated precipitation were determined in a small-scale experiment by gradually increasing the PEG concentration used for precipitation and analyzing the content of resuspended pellets on SDS-PAGE. For the 20S proteasome, PEG400 and concentrations between 20% (*v/v*) and 30% (*v/v*) were determined to be the optimal parameters for efficient precipitation. PEG precipitation is not selective but depends on several parameters like protein size, surface charge, pH, ionic strength, and other proteins present in the solution. Therefore, the precipitated pellet does not solely contain 20S proteasomes but also other proteins that co-precipitate (Figure 5B,C). 

The sucrose gradient allows for the separation of precipitated proteins and macromolecular complexes according to their size. By changing the slope of the linear sucrose density gradient different molecular sizes can be separated and their dispersion through the gradient can be adjusted. Suitable sucrose percentages, running speeds, and the duration of the centrifugation run are simulated by custom-developed software. A 10–30% (*w/v*) SW40 sucrose gradient run at 40,000 rpm for 16 h at 4 °C was determined to be optimal in the first step. 20S proteasome gradient fractions are identified by SDS-PAGE (Figure 5B), pooled, and re-precipitated with PEG to re-concentrate the sample. The changed composition of the pooled fractions compared to the cleared lysate results in an altered precipitation profile and therefore prevents the co-precipitation of protein impurities (Figure 5C). An additional sucrose gradient (Figure 5C) enables further purification of the 20S proteasome, adjustments of the linear gradient slope, running speed, and centrifugation time can yet again be performed to optimize the resolution in purification, or to limit dispersion in the gradient. For the 20S proteasome, a steeper sucrose gradient of 10–40 (*w/v*) resulted in better purity and higher concentrations. After recovering the 20S proteasome from the pooled fractions by PEG precipitation, SDS-PAGE validated the high purity of the purified 20S proteasomes. Yields of 20 mg from 300 mL starting material were reproducibly achieved, and the purification is completed in three days [73]. So far, several hundred protein crystal structures have been determined in a resolution range from 2.3 to 1.8 Å. This 20S proteasome purification scheme conceptually proved that the combination of PEG precipitation and sucrose density centrifugation is suitable for the purification of large macromolecular complexes. Moreover, all postulated requirements for the new purification protocol were met. 

### 4.2. Case Study 2: 20S Proteasome from Various Organisms and Recombinant Source

**Figure 6:**
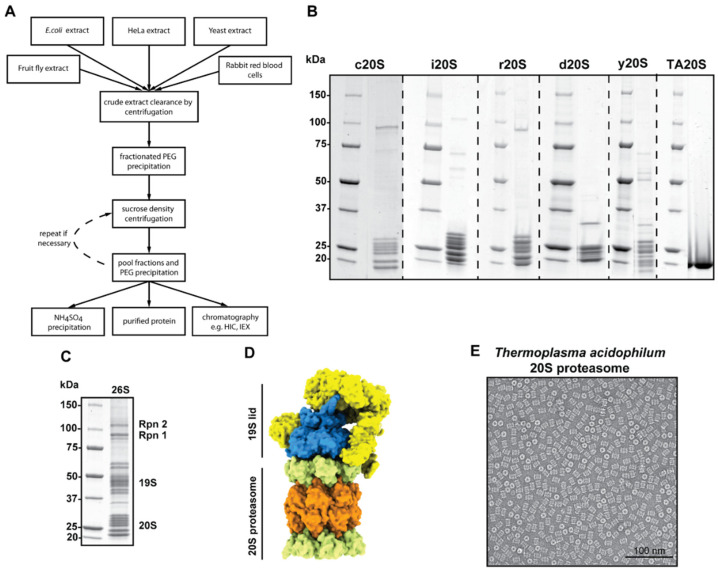
The human 20S proteasome purification protocol provides the framework to purify 20S proteasomes from different species (**A**). All purified 20S proteasomes are shown (**B**). SDS-PAGE of the purified 26S proteasome (**C**). The surface model representation of the 26S proteasome (PDB 6JWN) illustrates the complex architecture of the 26S proteasome (**D**). Negative stain EM analysis of the *Thermoplasma acidophilum* 20S proteasome shows the homogeneity of the sample (**E**).

The purification of the human 20S proteasome validated the use of PEG precipitation in combination with sucrose density gradient centrifugation. In a next step, we asked if the derived protocol was uniquely suited to the purification of human 20S proteasome or if it could be applied to other large macromolecular complexes as well. In a first step, we asked if 20S proteasomes from different sources, including 20S proteasomes from *Thermoplasma acidophilum* recombinantly expressed in *Escherichia coli*, 20S proteasomes from *Drosophila melanogaster* embryonic extract, 20S proteasomes from *Oryctolagus cuniculus* red blood cells, and 20S proteasomes from *Saccharomyces Cerevisiae* could be purified by adapting the purification protocol in case study 1 (Figure 6A). The different origins of samples imply variation in the composition of the starting material, which might affect the precipitation behavior of the 20S proteasomes. The preparation of the cleared lysate was adjusted to the properties of each organism. A heat denaturing step was introduced to the purification of the thermophilic, recombinantly expressed *T. acidophilum* 20S proteasomes allowing fast and efficient removal of most host proteins [74]. The protein concentration levels of the embryonic fruit fly extracts were diluted to match the protein concentrations measured for the starting material of the human 20S proteasome purification [75]. Furthermore, the buffer was adjusted to the same conditions as used for the purification of human 20S proteasomes. After hypotonic lysis of the rabbit red blood cells, the purification protocol was adopted from the human 20S proteasome purification. Yeast cells were resuspended in the optimized buffer before they were freeze-milled as beads for cell lysis. The following purification steps are common with those used for the purification of human 20S proteasomes: despite the different compositions of the cleared lysates, the PEG concentration needed for efficient precipitation of the 20S proteasome did not differ. For all different organisms pure 20S proteasomes were obtained (Figure 6B,E). Each of the purified 20S proteasomes were crystallized and high-resolution structures were determined which highlights the high sample quality provided by the PEG precipitation methodology. The extensive analysis of the PEG precipitation for the purification of 20S proteasomes from several different organisms suggests that the method is not restricted to a confined chemical space but can be adapted to the use for the purification of large macromolecular complexes in general. 

### 4.3. Case Study 3: 26S Proteasome from HeLa Cells

To assess the ability to purify larger and more fragile complexes, the 26S proteasome was purified from HeLa cells. The 26S proteasome (Figure 6D) consists of the 20S core particle and the 19S regulatory particle which in itself consists of 19 different subunits and has a size of 900 kDa. ATP-dependent protein degradation is mediated by the 26S proteasome [76,77]. Ubiquitinated substrates are recognized by the 19S lid, unfolded, and translocated in the proteolytic chamber—the 20S core particle [78]. Besides single-capped 26S proteasomes with a molecular weight of 1.5 MDa, 2.5 MDa double-capped 20S proteasomes also exist in cells [79]. From the 20S proteasome purification scheme described in case studies 1 and 2, it is evident that the 19S particle readily dissociates from the core particle and disintegrates as the 19S particle is absent. To avoid this, ATP was added to the lysate to stabilize the interaction between the 19S lid and the 20S core particle and prevent dissociation. Until precipitation of the 26S proteasome, all steps established for the purification of the 20S proteasome were transferred. Additionally, Proteoplex measurements of initial 26S proteasome preparations suggested that the buffer used for 20S proteasomes is also suitable for the 26S proteasome. The optimal PEG concentration for efficient precipitation of the 26S proteasome needed to be carefully adjusted. Empirical testing revealed that the 26S proteasome begins to precipitate at a concentration of 23% (*v/v*) PEG400. Above the concentration of 30% (*v/v*) PEG400 the 26S proteasome does not precipitate in significant amounts. Therefore, a PEG cut of 23–30% (*v/v*) was used to remove as many contaminations as possible and to recover most of the 26S proteasome from the lysate. Depletion of ATP, which disintegrates the 26S proteasome, was reduced by adding fresh ATP and an ATP regeneration system consisting of creatine kinase and creatine phosphate during resuspension of the precipitated 26S proteasome pellet. Running a linear sucrose gradient suitable to the size of the 26S proteasome did not result in a satisfying separation, instead, a double sucrose cushion composed of both a 20% (*w/v*) and 50% (*w/v*) sucrose layer was utilized. Better separation and higher concentration were achieved by the use of the sucrose cushion. Like for the 20S proteasomes, the 26S proteasome is recovered by PEG precipitation from the sucrose cushion fractions and loaded onto a linear sucrose gradient (10–40% (*w/v*)) after resuspension. PEG precipitation and another linear sucrose gradient (10–45% (*w/v*)) were necessary to obtain pure 26S proteasomes (Figure 6C). In the course of purification fresh ATP was always added to the resuspension buffer and was also included in the sucrose gradients to prevent its depletion. The quality of the purified 26S proteasome was checked by cryo-electron microscopy [80]. The number of dissociated particles is negligibly small. The purification of the 26S proteasome is a remarkable example of the purification of a large and fragile macromolecular complex. PEG precipitation preserves complex interactions of subunits which might be disrupted through small forces, like shearing forces, occuring during conventional chromatography. 

### 4.4. Case Study 4: Yeast Fatty Acid Synthase

**Figure 7:**
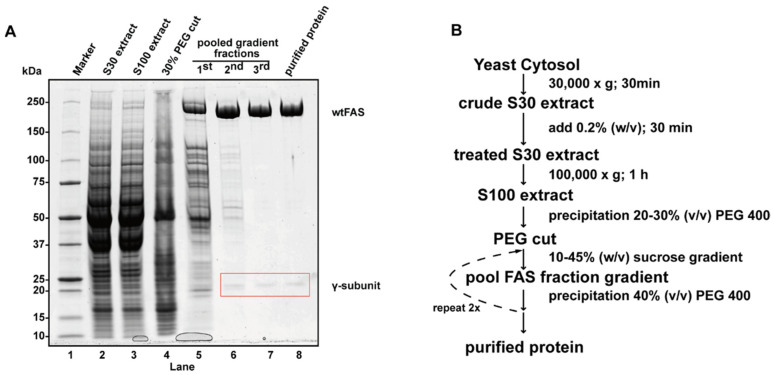
In the course of the yeast fatty acid purification, a sample after each purification step is analyzed on SDS-PAGE (**A**). The γ-subunit, which co-purifies with the yeast fatty acid synthase, is highlighted by a red rectangle. The complete purification protocol is shown as a concise flowchart (**B**).

In course of the yeast 20S proteasome purification, two high molecular protein bands were observed in fractions of the first sucrose gradient. These proteins are part of a large complex, as they are found in the bottom fractions of the sucrose gradient. Mass spectrometry revealed that they represented the yeast fatty acid synthetase. This yet again confirms that PEG precipitation is highly suitable for the purification of large macromolecular complexes, initial suggestions for the purification of other macromolecular machines can stem from the SDS-PAGE and MS/MS of sucrose density gradient fractions neighboring those of the complex of interest. An improved purification protocol was developed for the large-scale purification of the yeast fatty acid synthase which resembles the human 20S proteasome protocol (Figure 7B). The slope of the linear gradient needed to be adjusted according to the large size of 2.5 MDa and the buffer was optimized by Proteoplex, allowing the yeast fatty acid synthetase to be purified to high purity. Surprisingly, a small unknown protein of around 20 kDa co-purified with the yeast fatty acid synthetase. Further investigations established that the observed protein corresponds to a yet undiscovered, weakly bound yeast FAS γ-subunit that modulates its activity [47] (Chari et al., WO2020188074). Previously utilized purification protocols that employed higher ionic strength and chromatographic supports might have led to the dissociation of the FAS γ-subunit and explain why it had eluded discovery to date. Precipitation of macromolecular complexes by PEG preserves low-affinity protein interactions, which can be additionally stabilized by choosing suitable buffer conditions. 

### 4.5. Case Study 5: Combination of PEG Precipitation with Chromatographic Procedures

In the purification protocols described above, the combination of PEG precipitation and sucrose density centrifugation was presented as a stand-alone method for the purification of macromolecular complexes. However, several variations on a theme are conceivable and in this case study, we describe one which we have successfully applied. For the purification of the immunoproteasome, PEG precipitation and sucrose density gradients are supplemented by chromatographic procedures. The immunoproteasome is a subtype of the 20S proteasome in which the proteolytic active sites are replaced with immunoproteasome-specific subunits [81]. Immunoproteasomes are known to be involved in the generation of MHC-I associated peptides and are therefore part of the immune response [82]. Immunoproteasomes are constitutively expressed in hemopoietic cells and the expression can be modulated in non-immune cells by proinflammatory cytokines. Molt-4 cells induced for 72 h with interferon γ (IFN-γ) to achieve elevated immunoproteasome levels were used for the purification. The initial purification steps starting with the lysis of the cells and preparation of the crude extract were adopted from the human 20S proteasome purification. The protocols for the fractionated PEG precipitation and the sucrose gradient did not require any changes. Many impurities were removed in this step, as observed for the human 20S proteasome. As PEG precipitation is not specific for 20S proteasome subtypes, and separation on a sucrose gradient is not feasible due to the same size and shapes, the precipitated immunoproteasome and constitutive 20S proteasome were separated by hydrophobic interaction chromatography (HIC) [83]. The 20S proteasome sucrose gradient fractions were pooled and precipitated with PEG before dialysis against 1.7 M NH_4_SO_4_. This itself is a purification step, where salting-out at these ammonium sulfate concentrations aids in the removal of impurities. After the 20S proteasome subtypes were successfully separated on a large-pore monolithic HIC column, buffer exchange by initial dialysis, followed by PEG precipitation, instead of using the standard size exclusion chromatography (Figure 6B). Attempts to directly precipitate from high NH_4_SO_4_ concentrations resulted in insoluble protein precipitate. Thus, the incorporation of PEG precipitation in combination with sucrose density centrifugation is a versatile tool that can help in reducing chromatographic purification steps and can be implemented into established purification protocols. 

## 5. Considerations for the Incorporation into Biopharmaceutical Production Pipelines

Several groups have evaluated how PEG precipitation can be applied in a productive manner in the biopharmaceutical industry [84,85,86,87,88]. The clear advantages of PEG precipitation are the high efficiency and the cheap costs of the needed reagents. So far, the implementation of PEG precipitation in downstream processing is hindered by detailed models reliably explaining the PEG precipitation behavior of individual biopharmaceuticals and its dependency on environmental variation occurring during protein production [69]. The technical implementation of protein precipitation on a large-scale (separation of precipitates and supernatants) and the downstream processing of the precipitates are obstacles that need to be overcome before this method can be used in the biopharmaceutical industry on a large-scale. Moreover, the complete removal of residual PEG from the protein solution after PEG precipitation needs to be addressed when using the method in an industrial scale for the production of therapeutic agents. Yet, it should be emphasized that residual PEG does not compromise the integrity of the macromolecular complex and often increases the homogeneity of the samples for structure-based drug discovery (SBDD) campaigns. SBDD success inherently depends on high-quality samples for success. Great scientific advances were made to understand the parameters which influence protein precipitation by PEG [63,69,84,87,89]. Furthermore, the models to explain and predict PEG precipitation were gradually improved over the past years [64,66,67,90,91,92,93]. The focus of the latest studies was to develop PEG precipitation protocols to purify antibodies since they represent the protein class, which is purified most abundantly in the biopharmaceutical industry. While these impediments loom large for large-scale industrial processes, it has to be emphasized that drug discovery of agents targeting large macromolecular complexes remains a substantial bottleneck. Considering the capability of PEG precipitation in combination with sucrose gradients to purify precisely such macromolecular machines as shown for the presented case studies in mild conditions and high concentrations, the utility of this procedure in drug discovery is not to be neglected.

## 6. Outlook and Perspectives

In the future, further variations on a theme will benefit the methods presented in the case studies. These include studies that aim at describing the selectivity of PEG precipitation, and how this can be controlled or modified. New and other precipitation agents offer great potential to increase the selectivity for protein precipitation. Fractionation properties of different polymers like dextran, PVE, PVP, and NPE have already been investigated by Polson [63]. Their partially denaturing properties during protein precipitation and their high viscosities have prevented their useful application to date. However, modified PEG variants like PEGMA and other polymers like polyacrylates and polypropylene glycols may have advantageous fractionating properties. An interesting class of polymers in this regard are Jeffamines and Sokalans, which should be analyzed in closer detail (Chari et al. 2021U.S. Patent 2021017224) [94]. Small molecules can greatly influence precipitation characteristics. For example, ionic liquids are a recently described new class of small molecules altering the precipitation characteristics of proteins.

Aqueous two-phase systems with a subsequent protein precipitation step provides a potentially powerful method for protein selective protein purification [87]. Aqueous two-phase systems are based on the specific enrichment of a protein in one of the two phases or at the interphase. Known aqueous two-phase systems consist out of polymer–polymer, polymer–salt, alcohol–salt, ionic liquid–salt phases. By using functionalized or modified polymers the selective enrichment of a certain protein can be controlled. Another approach used by Maestro and colleagues was the introduction of a protein tag, allowing the affinity partitioning in an aqueous two-phase system [95]. Proteins can be enriched with the aqueous two-phase systems by centrifugal partitioning chromatography in large scale. Usually, the protein is recovered by chromatographic methods or ultrafiltration from the enriched phase. PEG precipitation could provide a quick and efficient approach to recover the partitioned proteins, which was already shown for monoclonal antibody purification [87].

There is increasing interest in purifying large macromolecular complexes to understand their functions and regulation. The established purification methods which use porous chromatographic media impose several disadvantages for the purification of macromolecular complexes. The disintegration of macromolecular complexes can be exacerbated by non-optimal purification conditions, shearing forces, and unspecific interactions with chromatographic media. While these limitations can be partially overcome by the use of large-pore monolithic chromatographic supports, or small, high-surface magnetic media, these limitations are entirely negligible for PEG precipitation. Several large macromolecular complexes have been purified by combining PEG precipitation with sucrose density centrifugation. Comparison with samples purified by conventional chromatographic methods has validated the high quality of the samples purified by PEG precipitation. Further developments and systematic analysis of the PEG precipitation technique will lead to the widespread application of PEG precipitation to purify large molecular complexes. 

## Data Availability

Not applicable.

## Referenced Patents

Chari, Ashwin and Stark, Holger and Schrader, Jil and Henneberg, Fabian. 2021. Method for the purification of biological macromolecular complexes. WO2017211775A1, filed 1 October 2020, and issued 21 January 2021. Stark, Holger and Chari, Ashwin and Haselbach, David and Kirves, Jan-Martin. 2016. Methods for analyzing biological macromolecular complexes and use thereof. WO2013034160, filed 6 September 2011, and issued 14 June 2016. Chari, Ashwin and Stark, Holger and Singh, Kashish and Graf, Benjamin. 2019. Fatty acid synthase, inhibitors and modifications thereof and its use. WO2020188074, filed 20 March 2019.
